# Draft Genome Sequence of Psychrotolerant *Shewanella* sp. Strain VB17, Isolated from Marine Intertidal Sediment near Virginia Beach, Virginia

**DOI:** 10.1128/MRA.00945-20

**Published:** 2020-11-19

**Authors:** Julia C. Clement, Paul D. Kastner, David A. Essig

**Affiliations:** a Department of Biology, Geneva College, Beaver Falls, Pennsylvania, USA; University of Southern California

## Abstract

We present the draft genome sequence of *Shewanella* sp. strain VB17, which was isolated from Virginia Beach, Virginia, intertidal sediment. The 5.2-Mb genome sequence of VB17 will be useful for studying the basis for seasonal low-temperature growth and eventual taxonomic classification of the species.

## Abstract (precis)

We present the draft genome sequence of *Shewanella* sp. strain VB17, which was isolated from Virginia Beach, Virginia, intertidal sediment. The 5.2-Mb genome sequence of VB17 will be useful to study the basis for seasonal low-temperature growth and eventual taxonomic classification of the species.

## ANNOUNCEMENT

Phylum-specific changes in bacterial abundance and diversity in marine environments are due to several factors, including seasonal temperature cycles (1, 2). To identify species that predominate in winter, we harvested bacteria from intertidal sediment (water temperature of 7°C, March 2019) offshore of Virginia Beach, Virginia. Sediment and pore water from a depth of 1 m were placed in a sterile plastic jar. Sediment was diluted (1:10) in phosphate-buffered saline (PBS) (pH 7.4), vortex mixed, spread onto Zobell marine agar 2216 plates, and incubated for 5 days at 4°C. Isolated colonies were picked, purified, and maintained at 4°C.

16S rRNA sequencing was performed by GENEWIZ (South Plainfield, NJ). A proprietary primer set was used to amplify regions V1 to V9 of the 16S gene to generate an ∼1,400-bp amplicon. Genomic DNA was extracted from colonies by NaOH lysis and used in PCR primer extension cycle sequencing with BigDye v3.1 (Applied Biosystems, Foster City, CA). Sequencing was performed on an Applied Biosystems 3730xl DNA analyzer. EzBioCloud (3) was used to generate pairwise alignments, and VB17 appeared to be a new species.

For genome sequencing, a pellet (1 × 10^7^ cells) was harvested from a log-phase culture in marine broth (25°C) by centrifugation for 5 min at 5,000 rpm. The pellet was resuspended in 1 ml of PBS (pH 7.4) with 10% glycerol and recentrifuged. The supernatant was aspirated to 50 μl, and the pellet was frozen. At ACGT, Inc. (Wheeling, IL), the pellet was resuspended in lysis buffer (10 mM Tris [pH 8.0], 50 mM EDTA, 1.5% SDS, 100 mg/ml RNase A, and 10 mg/ml lysozyme) and incubated at 37°C for 1 h. Subsequent steps in DNA purification were as described by Mayjonade et al. (4). The DNA concentration was determined using a Qubit fluorometer (Thermo Fisher Scientific, Waltham, MA) and size distribution by capillary electrophoresis (Agilent Technologies, Santa Clara, CA). An Illumina Nextera XT library was generated and sequenced using the MiSeq 300-cycle microkit (2 × 150 bp; Illumina, San Diego, CA). Sequence data analysis used default parameters unless otherwise specified. Total paired-end reads and yield were 2,648,103 reads and 794,430,900 bp, respectively. A Nanopore library (Oxford Nanopore Technologies, Oxford, UK) was prepared, barcoded, and sequenced in a MinION flow cell (v9.4, FLO-MIN106) using MinKNOW (v19.10.1) with Guppy (v3.2.6) as base caller. There were 2,505,014 reads totaling 13,434,593,130 bp (with an *N*_50_ value of 18,986 bp). The genome was assembled (Flye v2.5 [5]) and polished (Pilon v1.22 [6]), using Illumina reads, into two contigs (5,163,726 bp and 8,978 bp) with a total length of 5,172,704 bp (total coverage, 2,250×). The genome failed to circularize using Circlator (v1.5.5) (7).

The Prokaryotic Genome Annotation Pipeline (PGAP) (v4.12) (8) predicted 4,228 protein-coding genes and 131 RNA genes. The Genome-to-Genome Distance Calculator (formula 2) (9) considered VB17 a species distinct from the closely related species Shewanella hanedai (22.00%) and Shewanella woodyi (21.70%), based on a same-species threshold of 70.00% sequence identity.

### Data availability.

This whole-genome shotgun project is in GenBank under accession number JABRVS000000000.1. The first version is described in this paper. The SRA accession numbers for the MinION and Illumina libraries are SRR11858603 and SRR11858604, respectively.
